# Real world practice of postoperative radiotherapy for patients with completely resected pIIIA-N2 non-small cell lung cancer: a national survey of radiation oncologists in China

**DOI:** 10.1186/s13014-023-02208-5

**Published:** 2023-01-25

**Authors:** Shujie Zhou, Yirui Zhai, Kaikai Zhao, Yu Men, Xiangjiao Meng, Zhouguang Hui

**Affiliations:** 1 Department of Radiation Oncology, Shandong Cancer Hospital Affiliated to Shandong University, Jinan, 250117 Shandong China; 2 Department of Radiation Oncology, National Cancer Center/National Clinical Research Center for Cancer/Cancer Hospital, Chinese Academy of Medical Sciences and Peking Union Medical College, Beijing, 100021 China; 3 Department of VIP Medical Services, National Cancer Center/National Clinical Research Center for Cancer/Cancer Hospital, Chinese Academy of Medical Sciences and Peking Union Medical College, Beijing, 100021 China; 4 Department of Radiation Oncology, Shandong Cancer Hospital and Institute, Shandong First Medical University and Shandong Academy of Medical Sciences, Jinan, China

**Keywords:** PORT (postoperative radiotherapy), NSCLC (non-small cell lung cancer), Expert opinions, Risk factors

## Abstract

**Background:**

Results from Lung ART and PORT-C trials suggest that postoperative radiotherapy (PORT) cannot routinely be recommended as standard treatment in completely resected pIIIA-N2 NSCLC patients, but their effects on the real-world practice of PORT in China remain unclear.

**Methods:**

A national cross-section survey was conducted by using an online survey service. Participants were voluntarily recruited using a river sampling strategy. A link to the survey was posted on websites of radiation oncologist associations and tweets from public WeChat accounts. The survey collected the real names of participants to ensure that they were board-certified radiation oncologists.

**Results:**

A total of 484 radiation oncologists were included with a median age of 40 years (IQR, 35–47). A total of 377 (77.9%) participants were male, and 282 (58.1%) had more than 10 years of clinical experience practicing thoracic radiotherapy. Before Lung ART and PORT-C trials were published, 313 (64.7%) respondents recommended PORT, 11 (2.3%) did not recommend it, and 160 (33.1%) reported that they made decisions based on risk factors. After the presentation of two trials, only 42 (8.7%) did not recommend PORT, while 108 (22.3%) recommended it, and 334 (69.0%) made decisions based on risk factors. The five most commonly considered risk factors among these 334 respondents were as follows: nodal extracapsular extension, the highest lymph node (LN) station involved, the number of dissected mediastinal LN stations, the number of positive mediastinal LN stations, and surgical approaches. In addition, the majority of all 484 respondents recommended a total dose of 50 Gy, lung stump + ipsilateral hilus + regions containing positive LNs as the targeted region, lung V20 < 25%, and heart V30 < 40% as dose constraints for PORT.

**Conclusion:**

Most Chinese radiation oncologists recommended PORT for completely resected IIIA-N2 NSCLC patients based on risk factors, especially status of LN station.

**Supplementary Information:**

The online version contains supplementary material available at 10.1186/s13014-023-02208-5.

## Background

Lung cancer has one of the highest global incidence rates as well as the highest global mortality rate among all malignancies [1]. It is estimated that nearly one-third of patients with non-small cell lung cancer (NSCLC) are stage III at diagnosis, and surgical resection-based strategies are considered the primary treatment for stage IIIA NSCLC patients, with a selection rate of 76.2% [2, 3]. There is still a significant risk of local–regional recurrence (LRR) and distant metastasis in patients with pN2 stage who receive surgical resection alone, which implies that those patients need adjuvant treatment [4]. The history of the utilities of postoperative radiotherapy (PORT) in NSCLC of stage IIIA-N2 is complex and variable due to inconsistent results from different studies.

In 1998, a meta-analysis published in Lancet suggested that PORT was associated with worse survival in NSCLC patients with early-stage disease (pI-II or pN0-1), which made it was abandoned in the treatment of these low-risk patients. However, the role of PORT in pIIIA-N2 patients was uncertain [5]. Additionally, it should be noted that patients included in this study did not receive systemic treatment with outdated radiotherapy techniques, leading to a high risk of distant relapse and toxicity [5].

In 2006, the subgroup analysis of ANITA study demonstrated an obvious increase in survival for pN2 patients with complete resection who received PORT, which facilitated further study evaluating the role of PORT on completely resected pIIIA-N2 patients [6]. These positive results promoted the conduction of prospective clinical trials. Furthermore, a series of retrospective studies showed that PORT was associated with an additional OS advantage in the subgroup of pN2 patients who had complete resection and were treated with adjuvant chemotherapy [7–9]. During that time, PORT was officially recommended by the guidelines and was thus delivered more routinely. Prospective studies of PORT are ongoing. Recently, two large phase 3 randomized clinical trials, the Lung ART and PORT-C trial, have demonstrated that administering PORT to completely resected pIIIA-N2 NSCLC patients does not lead to significant improvement in disease-free survival (DFS) [10, 11].

Nevertheless, a marginal benefit for 3-year DFS was observed in the per-protocol (PP) population who received PORT in the PORT-C trial, and prolonged median DFS was also reported in the Lung ART [11]. Additionally, almost all recent studies on this topic have affirmed the efficacy of PORT for decreasing LRR. These means that there are potential populations that may benefit from PORT and should be correctly identified. Meanwhile, a considerable proportion of patients still experience LRR and distant metastasis, especially those with pN2 stage, which is associated with poor overall survival (OS) for patients with NSCLC [4]. Therefore, it is not rational strategy to completely discard PORT in completely resected pIIIA-N2 patients. Hence, the current survey aims to investigate opinions among radiation oncologists on the following matters. How were decisions made before and after the publication of the Lung ART and PORT-C trial? Which patients are at high risk of relapse after PORT? How can PORT be best implemented in clinical practice for treating completely resected pIIIA-N2 NSCLC patients?

## Material and methods

The survey was administered using a voluntary survey by using a professional online survey service, Questionnaire Star (https://www.wjx.cn), between February 8, 2022, and April 30, 2022. In total, 41 questions assessed demographics, clinical decision-making related to PORT before and after the publication of the Lung ART and PORT-C trial, respondents’ characteristics, risk factors and radiotherapy-related factors influencing the implementation of PORT in completely resected pIIIA-N2 NSCLC patients. The details of the questions are listed in the Additional file 1. A link to the survey was posted on the websites of radiation oncologists’ associations and tweets from public WeChat accounts. All respondents were professionally trained thoracic radiation oncologists. Participants had to provide their real name, but we only used this information to confirm their eligibility. Descriptive analyses were performed by using frequency distributions or rates. Statistical analysis of the data was performed using the chi-square test.

## Results

### Respondent characteristics

Ultimately, 484 Chinese radiation oncologists voluntarily completed the online survey across 29 provinces, autonomous regions and municipalities of mainland China. The median age of respondents was 40 years old [interquartile range (IQR), 35–47], and 77.9% were male. Clinical experience was defined as years practicing thoracic radiation. The majority (58.1%) of respondents had more than 10 years of clinical experience, and 52.2% had a senior professional title. According to 2021 per capita gross domestic product (GDP) in mainland China, 68.1% of respondents came from intermediately developed regions [12]. Respondents came from 34 oncology specialty hospitals and 191 general hospitals, and most of them worked in general hospitals (71.1%) with large-scale tertiary class A hospital grade (74.4%). A majority of them could implement intensity-modulated radiotherapy (IMRT) or more advanced techniques (68.2%) to treat their patients. In addition, 74.4% and 73.1% of respondents reported some knowledge about the Lung ART and PORT-C trial, respectively (Table 1).

**Table 1:** Demographic characteristics of all respondents

Characteristic	No. (%)
*Age (years)*	
Median	40
IQR	35–47
*Gender*	
Male	377 (77.9)
Female	107 (22.1)
*Clinical experience*	
1–5 years	82 (16.9)
6–10 years	121 (25.0)
11–20 years	163 (33.7)
≥ 21 years	118 (24.4)
*Professional titles*	
Senior title	253 (52.2)
Intermediate title	176 (36.4)
Junior title	55 (11.4)
*Economic levels*	
Underdeveloped	54 (11.2)
Intermediately developed	330 (68.1)
Developed	100 (20.7)
*Hospital type*	
Comprehensive hospital	347 (71.7)
Cancer specialty hospital	137 (28.3)
*Hospital rank*	
Tertiary class A hospital	358 (74.4)
Tertiary class B hospital	67 (13.8)
Secondary hospital	57 (11.8)
*PORT techniques*	
2D-RT	2 (0.4)
3D-CRT	42 (8.7)
IMRT	330 (68.2)
VMAT	107 (22.1)
TOMO	3 (0.6)
*Knowledge of LungART trial*	
No	124 (25.6)
Yes	360 (74.4)
*Knowledge of PORT-C trial*	
No	130 (26.9)
Yes	354 (73.1)

*IQR* Interquartile range, *PORT* Postoperative radiotherapy, *2D-RT* 2D radiation therapy, *3D-CRT* 3D-conformal radiation therapy, *IMRT* Intensity-modulated radiation therapy, *VMAT* Volumetric modulated arc therapy, *TOMO* tomotherapy

### The influence of the two RCTs

**Fig. 1:**
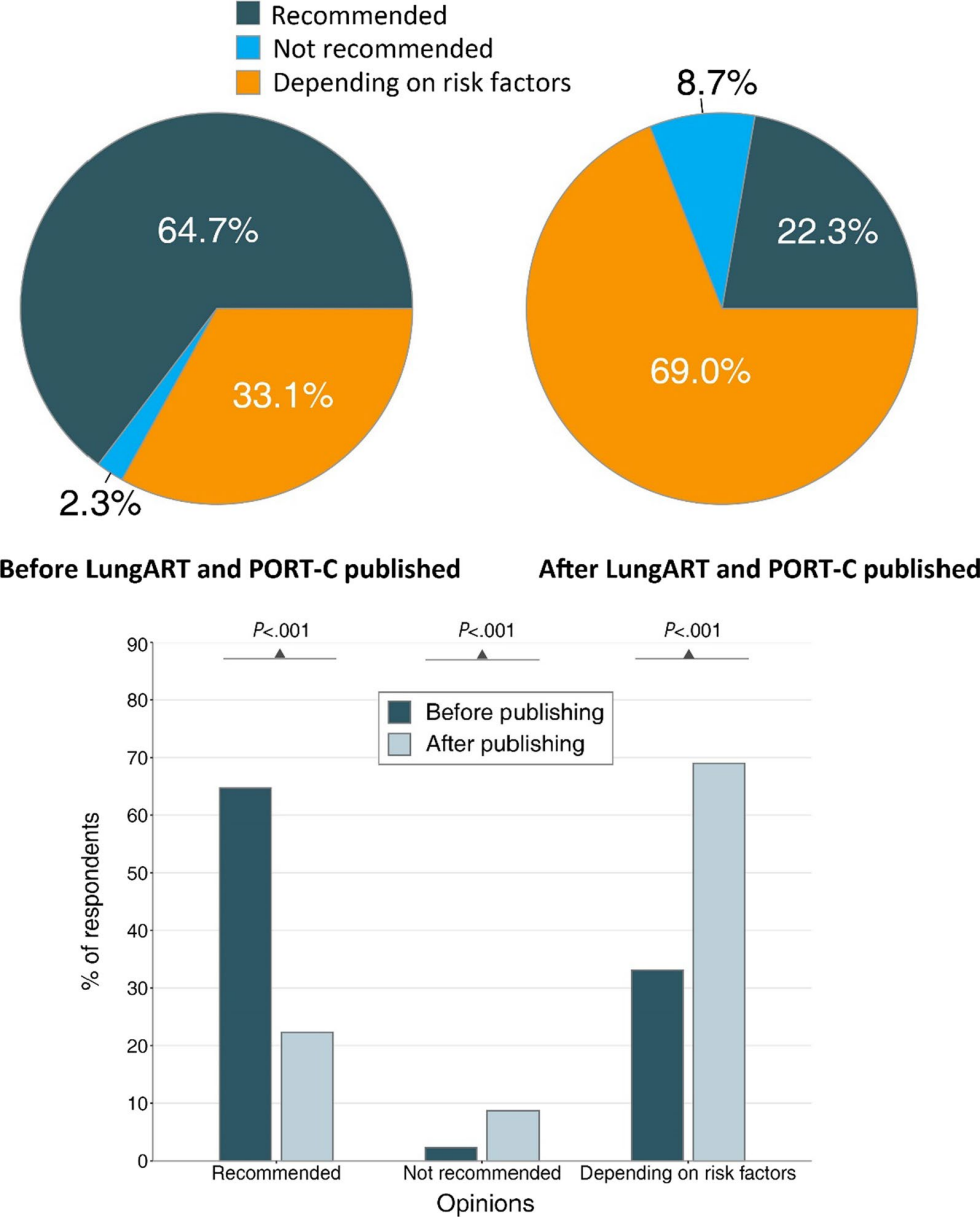
Change of Respondents’ Opinions on Postoperative Radiotherapy (PORT) before and after Lung ART and PORT-C Published

Before the Lung ART and PORT-C trial were published, the majority of respondents (64.7%) regularly recommended PORT for completely resected IIIA-N2 NSCLC, 2.3% did not recommend it, and 33.1% said they made the decision based on patients’ individual risk factors. After the two trials were published, the proportion of respondents regularly recommending PORT decreased by 22.3%. The proportion of respondents who did not routinely recommend PORT increased to 8.7%, and 69.0% of respondents made decisions based on individual risk factors (Fig. 1). Additionally, respondents were grouped based on their clinical experiences and their knowledge of the two trials. Overall, findings from the survey were largely congruent with regard to choice preferences and no major differences were observed among all experience groups (Table 2). The proportion of respondents regularly recommending PORT decreased by 43.9% for respondents who were not knowledgeable about either of trials after the publishing of trials, while it was 15.7% for those knowledgeable about them. The proportion of not recommending PORT did not change among respondents not knowledgeable about trials, whereas it grew from 1.9 to 10.3% among respondents knowing about them. The proportions regarding respondents made decisions based on individual risk factors were 52.6% and 74.1% in the two groups after publishing of two trials, respectively (Table 3). These results suggested that whether radiation oncologists knew the two RCTs did affect what decisions of PORT that they made.

**Table 2:** Respondents’ opinions on postoperative radiotherapy (PORT) before and after lung ART and PORT-C published are grouped based on their clinical experiences

Options	Clinical experience (years)—N (%)				
	All (n = 484)	1–5 (n = 82)	6–10 (n = 121)	11–20 (n = 163)	≥ 21 (n = 118)
*Before published*					
Recommended	313 (64.7)	50 (61.0)	80 (66.1)	104 (63.8)	79 (66.9)
Not recommended	11 (2.3)	5 (6.1)	1 (0.8)	3 (1.8)	2 (1.7)
Depending on risk factors	160 (33.1)	27 (32.9)	40 (33.1)	56 (34.4)	37 (31.4)
*After published*					
Recommended	108 (22.3)	20 (24.4)	25 (20.7)	36 (22.1)	27 (22.9)
Not recommended	42 (8.7)	7 (8.5)	12 (9.9)	12 (7.4)	11 (9.3)
Depending on risk factors	334 (69.0)	55 (67.1)	84 (69.4)	115 (70.6)	80 (67.8)

**Table 3:** Respondents’ opinions on postoperative radiotherapy (PORT) before and after lung ART and PORT-C published are grouped based on their knowledge of trials

Options	Knowledge of LUNG-ART and PORT-C trial—N (%)		
	All (n = 484)	Not knowledgeable about either of trials (n = 114)	Knowledgeable about either of trials (n = 370)
*Before published*			
Recommended	313 (64.7)	65 (57.0)	248 (67.0)
Not recommended	11 (2.3)	4 (3.5)	7 (1.9)
Depending on risk factors	160 (33.1)	45 (39.5)	115 (31.1)
*After published*			
Recommended	108 (22.3)	50 (43.9)	58 (15.7)
Not recommended	42 (8.7)	4 (3.5)	38 (10.3)
Depending on risk factors	334 (69.0)	60 (52.6)	274 (74.1)

### Risk factors

**Fig. 2:**
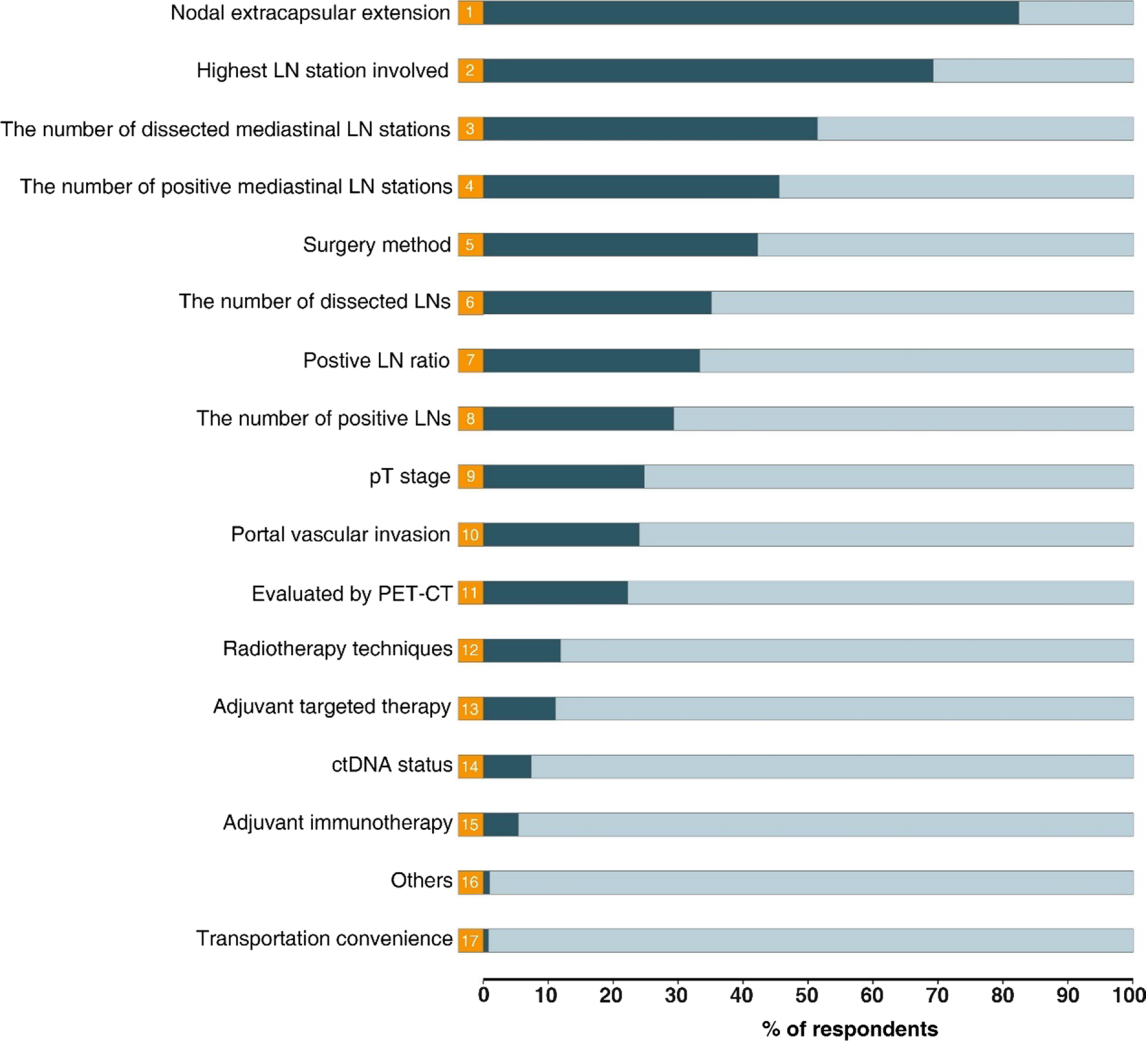
Proportions of Selection of Risk Factors Affecting Decision-making of Postoperative Radiotherapy (PORT). Respondents were asked to select the 5 most significant risk factors among 16 provided risk factors. If a risk factor was not provided as an option, respondents could select the ‘Other’ option and reported it using plain text. Abbreviation: *LN* Lymph node, *PET-CT* Positron emission tomography with computed tomography, *ctDNA* Circulating tumor DNA

Furthermore, we wanted to determine which risk factors play key roles in the decision-making related to PORT among the 334 respondents who replied ‘based on individual risk factors. We examined 16 different risk factors that may affect the decision-making related to PORT. These risk factors were selected based on a literature review and expert clinician opinions, and respondents were asked to select the top five most important risk factors. If a particular risk factor was not provided as an option, respondents were able to select the ‘Other’ option and then type in a risk factor. The five most commonly considered risk factors were as follows: nodal extracapsular extension (83.5%), highest lymph node (LN) station involved (71.6%), the number of dissected mediastinal LN stations (53.0%), the number of positive mediastinal LN stations (45.5%), and the choice of surgical approaches (42.2%) (Fig. 2).

**Fig. 3:**
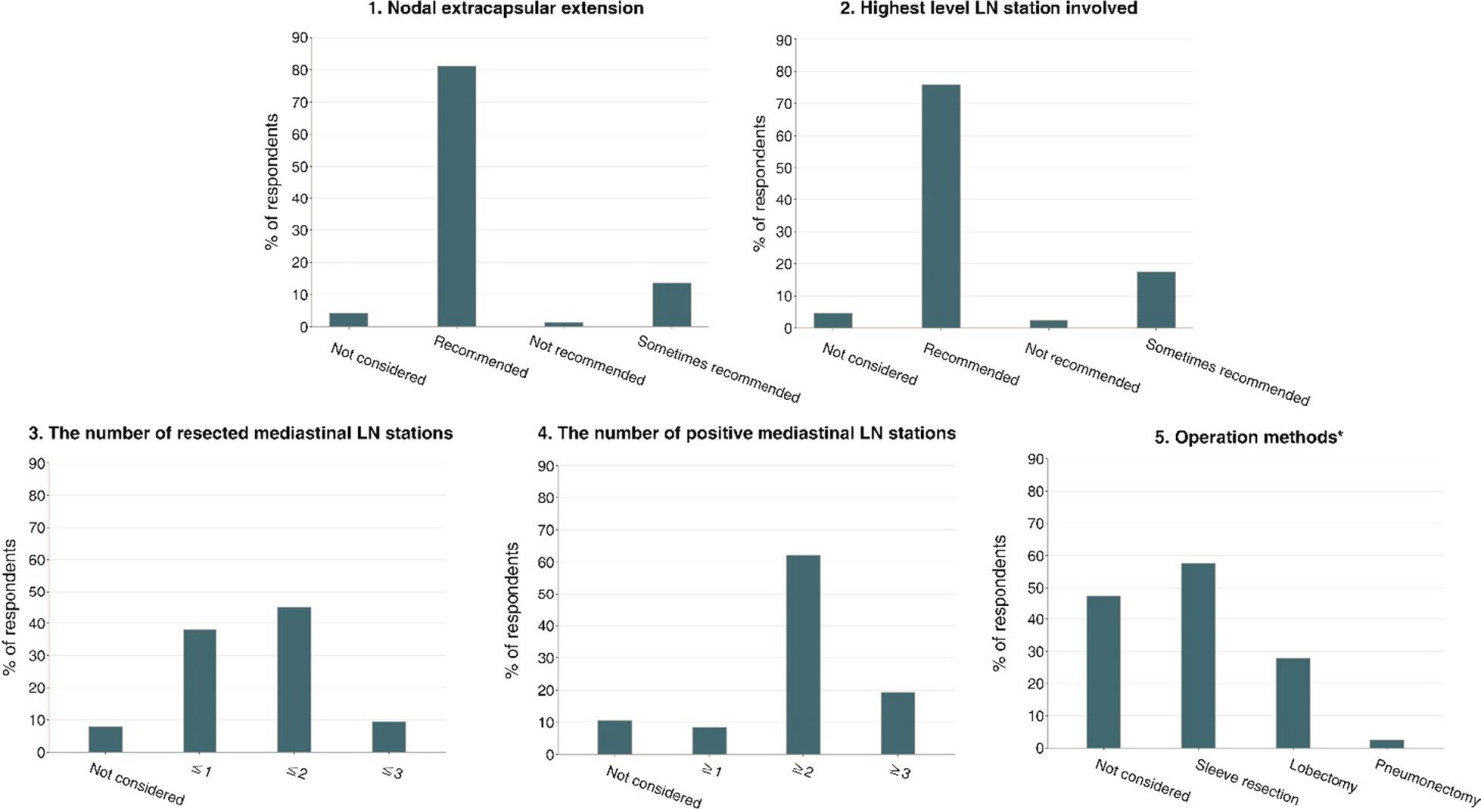
Selection details of top five risk factors affecting decision-making of postoperative radiotherapy (PORT). Abbreviation: *LN* lymph node. *Multiple selection

Then, we presented the details of the top five risk factors affecting the decision-making related to PORT (Fig. 3). If nodal extracapsular extension was present, the majority of respondents (81.1%) reported that they recommended PORT for patients with this key risk factor. In addition, approximately three-quarters of respondents recommended PORT for patients who had the highest LN station involvement. Next, we wanted to determine the specific values of several risk factors that might prompt respondents to recommend PORT (Table 4). Most of the radiation oncologists (88.8%) reported that dissected mediastinal LN stations strongly influenced their decision. In total, 38.5% of the radiation oncologists chose PORT if ≤ 3 lymph node stations were dissected. Additionally, 41.0% and 9.3% of the radiation oncologists recommended PORT for patients with ≤ 2 and ≤ 1 dissected lymph node stations, respectively. The proportions of respondents who recommended PORT for patients with ≤ 3 and ≤ 2 dissected lymph nodes were similar. For the number of positive mediastinal LN stations, the majority of respondents (62%) selected more than or equal to 2 as the cutoff for recommending PORT. The proportions of surgical approaches chosen (multiple selection) by respondents were as follows: pneumonectomy (2.4%), lobectomy (27.8%), sleeve lobectomy 57.5%), and not a consideration (47.0%) (Table 4). This suggests that more than half of respondents thought that patients who underwent sleeve lobectomy were more likely to relapse and might require PORT.

**Table 4:** Questions and responses of risk factors among 334 respondents who reported recommending postoperative radiotherapy (PORT) are grouped based on respondents’ clinical experience

Risk factors	Clinical experience (years)—responders N (%)				
	All (n = 334)	1–5 (n = 44)	6–10 (n = 84)	11–20 (n = 115)	≥ 21 (n = 91)
*Nodal extracapsular extension*					
Recommend	271 (81.1)	31 (70.5)	72 (85.7)	97 (84.3)	71 (78.0)
Sometimes recommend	45 (13.5)	7 (15.9)	10 (11.9)	14 (12.2)	14 (15.4)
Not recommend	4 (1.2)	1 (2.3)	3 (3.6)	1 (0.9)	1 (1.1)
Not considered	14 (4.2)	5 (11.4)	1 (1.2)	3 (2.6)	5 (5.5)
*Highest level LN station involved*					
Recommend	253 (75.7)	34 (77.3)	68 (81.0)	88 (76.5)	63 (69.2)
Sometimes recommend	58.1 (17.4)	4 (9.1)	13 (15.5)	19 (16.5)	22 (24.2)
Not recommend	8 (2.4)	2 (4.5)	0 (0.0)	2 (1.7)	4 (4.4)
Not considered	15 (4.5)	4 (9.1)	3 (3.6)	6 (5.2)	2 (2.2)
*The number of resected LNs*					
≤ 10	164 (49.1)	20 (45.5)	43 (51.2)	62 (53.9)	39 (42.9)
≤ 11	2 (0.6)	2 (4.5)	0 (0.0)	0 (0.0)	0 (0.0)
≤ 12	48 (14.4)	9 (20.5)	12 (14.3)	10 (8.7)	17 (18.7)
≤ 13	2 (0.6)	0 (0.0)	0 (0.0)	2 (1.7)	0 (0.0)
≤ 14	6 (1.8)	2 (4.5)	2 (2.4)	2 (1.7)	0 (0.0)
≤ 15	22 (6.6)	3 (6.8)	4 (4.8)	9 (7.8)	6 (6.6)
≤ 16	7 (2.1)	2 (4.5)	2 (2.4)	3 (2.6)	0 (0.0)
Not considered	83 (24.9)	6 (13.6)	21 (25.0)	27 (23.5)	29 (31.9)
*The number of resected mediastinal LN stations*					
≤ 1	26 (7.8)	8 (18.2)	6 (7.1)	5 (4.3)	7 (7.7)
≤ 2	127 (38.0)	15 (34.1)	31 (36.9)	46 (40.0)	35 (38.5)
≤ 3	150 (44.9)	20 (45.5)	33 (39.3)	54 (47.0)	43 (47.3)
Not considered	31 (9.3)	1 (2.3)	14 (16.7)	10 (8.7)	6 (6.6)
*The number of positive LNs*					
≥ 1	10 (3.0)	2 (4.5)	1 (1.2)	3 (2.6)	4 (4.4)
≥ 2	25 (7.5)	2 (4.5)	9 (10.7)	8 (7.0)	6 (6.6)
≥ 3	103 (30.8)	17 (38.6)	23 (27.4)	38 (33.0)	25 (27.5)
≥ 4	43 (12.9)	7 (15.9)	11 (13.1)	13 (11.3)	12 (13.2)
≥ 5	14 (4.2)	1 (2.3)	3 (3.6)	7 (6.1)	3 (3.3)
≥ 6	6 (1.8)	1 (2.3)	0 (0.0)	3 (2.6)	2 (2.2)
Not considered	133 (39.8)	14 (31.8)	37 (44.0)	43 (37.4)	39 (42.9)
*The number of positive mediastinal LN stations*					
≥ 1	28 (8.4)	6 (13.6)	7 (8.3)	9 (7.8)	6 (6.6)
≥ 2	207 (62.0)	22 (50.0)	56 (66.7)	72 (62.6)	57 (62.6)
≥ 3	64 (19.2)	11 (25.0)	12 (14.3)	24 (20.9)	17 (18.7)
Not considered	35 (10.5)	5 (11.4)	9 (10.7)	10 (8.7)	11 (12.1)
*Positive LN ratio*					
≥ 10%	18 (5.4)	4 (9.1)	3 (3.6)	5 (4.3)	6 (6.6)
≥ 20%	61 (18.3)	8 (18.2)	15 (17.9)	24 (20.9)	14 (15.4)
≥ 30%	91 (27.2)	11 (25.0)	19 (22.6)	35 (30.4)	26 (28.6)
≥ 40%	12 (3.6)	1 (2.3)	3 (3.6)	5 (4.3)	3 (3.3)
≥ 50%	73 (21.9)	13 (29.5)	21 (25.0)	26 (22.6)	13 (14.3)
Not considered	79 (23.7)	7 (15.9)	23 (27.4)	20 (17.4)	29 (31.9)
*Surgical approaches*^a^					
Pneumonectomy	8 (2.4)	2 (4.5)	1 (1.2)	1 (0.9)	4 (4.4)
Lobectomy	93 (27.8)	10 (22.7)	21 (25.0)	35 (30.4)	27 (29.7)
Sleeve lobectomy	192 (57.5)	21 (47.7)	52 (61.9)	75 (65.2)	44 (48.4)
Not considered	157 (47.0)	21 (47.7)	34 (40.5)	52 (45.2)	50 (54.9)
*Margin distance*					
≤ 0.5 cm	120 (35.9)	13 (29.5)	30 (35.7)	36 (31.3)	41 (45.1)
≤ 1 cm	93 (27.8)	14 (31.8)	23 (27.4)	35 (30.4)	21 (23.1)
≤ 1.5 cm	24 (7.2)	3 (6.8)	9 (10.7)	10 (8.7)	2 (2.2)
≤ 2 cm	57 (17.1)	7 (15.9)	16 (19.0)	24 (20.9)	10 (11.0)
Not considered	40 (12.0)	7 (15.9)	6 (7.1)	10 (8.7)	17 (18.7)
*pT stage*					
≥ 1b	6 (1.8)	3 (6.8)	1 (1.2)	2 (1.7)	0 (0.0)
≥ 1c	2 (0.6)	1 (2.3)	1 (1.2)	0 (0.0)	0 (0.0)
≥ 2a	25 (7.5)	9 (20.5)	6 (7.1)	4 (3.5)	6 (6.6)
≥ 2b	48 (14.4)	4 (9.1)	11 (13.1)	22 (19.1)	11 (12.1)
≥ 3	124 (37.1)	17 (38.6)	30 (35.7)	40 (34.8)	37 (40.7)
≥ 4	53 (15.9)	5 (11.4)	14 (16.7)	21 (18.3)	13 (14.3)
Not considered	76 (22.8)	5 (11.4)	21 (25.0)	26 (22.6)	24 (26.4)
*Carrying driver mutations*^a^					
No adjuvant treatment	141 (42.2)	13 (29.5)	41 (48.8)	45 (39.1)	42 (46.2)
Chemotherapy	122 (36.5)	13 (29.5)	31 (36.9)	44 (38.3)	34 (37.4)
Targeted therapy	100 (29.9)	17 (38.6)	23 (27.4)	28 (24.3)	32 (35.2)
Chemotherapy + targeted therapy	67 (20.1)	11 (25.0)	12 (14.3)	26 (22.6)	18 (19.8)
Always Recommend	41 (12.3)	7 (15.9)	7 (8.3)	15 (13.0)	12 (13.2)
Always not recommend	41 (12.3)	7 (15.9)	8 (9.5)	15 (13.0)	11 (12.1)
*PD-L1 expression positive*^a^					
No adjuvant treatment	144 (43.1)	11 (25.0)	40 (47.6)	51 (44.3)	42 (46.2)
Chemotherapy	138 (41.3)	16 (36.4)	29 (34.5)	55 (47.8)	38 (41.8)
Immunotherapy	74 (22.2)	16 (36.4)	19 (22.6)	22 (19.1)	17 (18.7)
Chemotherapy + Immunotherapy	86 (25.7)	9 (20.5)	24 (28.6)	29 (25.2)	24 (26.4)
Always recommend	45 (13.5)	6 (13.6)	10 (11.9)	15 (13.0)	14 (15.4)
Always not recommend	31 (9.3)	6 (13.6)	7 (8.3)	10 (8.7)	8 (8.8)

*LNs* Lymph nodes, *PD-L1* Programmed cell death ligand-1

^a^Multiple selection

Excluding LN-related risk factors, pT stage was the most important consideration, and most respondents (37.1%) recommended PORT for patients with a pT stage greater than or equal to 3 (Table 4). Circulating tumor DNA (ctDNA) is receiving increasing attention as a useful biomarker to detect minimal residual disease (MRD) following surgical resection, which helps to identify high-risk patients [13]. However, in our study, we found that ctDNA was considered a low priority, with the bottom third of risk factors ranking. Given that targeted therapies and immunotherapy have been approved in the adjuvant setting for patients with stage III disease, the role of PORT in those patients has not been previously reported. In our study, approximately 30% of respondents thought patients with driver mutations treated with adjuvant targeted therapy might require PORT, and a slightly higher proportion (36.5%) thought that adjuvant chemotherapy was the most appropriate treatment. For patients with positive PD-L1 expression, 22.2% of respondents considered the application of PORT if immunotherapy was used, while nearly twice as many respondents (41.3%) suggested PORT if chemotherapy was used (Table 4). These data suggest that respondents tended to recommend PORT for patients treated with adjuvant targeted therapy more often than for patients treated with adjuvant immunotherapy.

### Radiotherapy delivery

Next, we investigated current PORT practices, including dose, clinical targeted volume (CTV), prophylactic treatment, and dose constraints, among all 484 respondents (Table 5). The total doses in the Lung ART and PORT-C trial were 54 Gy and 50 Gy, respectively, which is not consistent, and thus, we wanted to examine these doses in clinical practice. The majority of respondents (78.9%) reported that the total dose they used was 50 Gy. Lung stump + ipsilateral hilus + regions containing positive LNs composed the most frequent CTV used by respondents (58.5%). The vast majority of respondents (91.6%) considered that supraclavicular regions should be treated with prophylactic radiotherapy if patients had positive mediastinal LN at level 2 and required PORT. In addition, 75.2% reported that the contralateral mediastinum should be treated with prophylactic radiotherapy if patients had a positive LN ratio of 100% at level 7. We also investigated the volume (V) percentage of the lung and heart receiving a specific gray dose. If patients underwent lobectomy for a single lung lobe, nearly half of the respondents (44.8%) reported that V20 was less than 25% in their medical institutions. For the heart, 90.3% of respondents reported the dose constraint of heart V30, and its value was mostly less than 40%. Additionally, 50.4% claimed a dose constraint of less than 30% heart V40 (Table 5).

**Table 5:** Questions and responses of radiotherapy-related details are grouped based on clinical experience among all respondents

Radiotherapy-related details	Clinical experience (years)---Responders N (%)				
	All (n=484)	1–5 (n=82)	6–10 (n=121)	11-20 (n=163)	≥ 21 (n=118)
*Total dose*					
50 Gy	382 (78.9)	56 (68.3)	98 (81.0)	133 (81.6)	95 (80.5)
54 Gy	99 (20.5)	25 (30.5)	22 (18.2)	29 (17.8)	23 (19.5)
Others	3 (0.6)	1 (1.2)	1 (0.8)	1 (0.6)	0 (0.0)
*Targeted region closest to clinical practice*^*a*^					
Lung stump + ipsilateral hilus + regions containing positive LNs	283 (58.5)	42 (51.2)	62 (51.2)	99 (60.7)	80 (67.8)
Lung stump + ipsilateral mediastinum ± ipsilateral supraclavicular LNs^b^	238 (49.3)	52 (63.4)	64 (52.9)	75 (46.3)	47 (39.8)
Lung stump + ipsilateral mediastinum ± contralateral upper mediastinum ^c^	132 (27.3)	22 (26.8)	26 (21.5)	49 (30.1)	35 (29.7)
*Prophylactic radiotherapy for stump*^*a*^					
Surgical margins were near to tumor margins	375 (77.5)	56 (68.3)	89 (73.6)	131(80.4)	99 (83.9)
Central type	214 (44.2)	27 (32.9)	51 (42.1)	76 (46.6)	60 (50.8)
Any condition	102 (21.1)	21 (25.6)	28 (23.1)	32 (19.6)	21 (17.8)
*Lung V20*					
<20%	173 (35.7)	33 (40.2)	43 (35.5)	51 (31.3)	46 (39.0)
<25%	217 (44.8)	33 (40.2)	51 (42.1)	79 (48.5)	54 (45.8)
<30%	87 (18.0)	16 (19.5)	24 (19.8)	32 (19.6)	15 (12.7)
Others	7 (1.4)	0 (0.0)	3 (2.5)	1 (0.6)	3 (2.5)
*Heart dose constraint *^*a, c*^					
No limitation	19 (3.9)	10 (12.2)	4 (3.3)	1 (0.6)	4 (3.4)
Yes, Heart V30	437 (90.3)	68 (82.9)	107 (88.4)	152 (93.3)	110 (93.2)
<30%	71 (14.7)	7 (8.5)	18 (14.9)	23 (14.1)	23 (20.9)
<35%	45 (9.3)	6 (7.3)	5 (4.1)	17 (11.2)	17 (15.5)
<40%	280 (57.9)	43 (52.4)	75 (62.0)	100 (65.8)	62 (56.4)
Other	41 (8.5)	12 (14.6)	9 (7.4)	12 (7.9)	8 (7.3)
Yes, Heart V40	244 (50.4)	30 (36.6)	60 (49.6)	89 (54.6)	65 (55.1)
<20%	14 (2.9)	1 (1.2)	4 (3.3)	3 (1.8)	6 (5.1)
<25%	13 (2.7)	2 (2.4)	1 (0.8)	6 (3.7)	4 (3.4)
<30%	207 (42.8)	26 (31.7)	53 (43.8)	76 (46.6)	52 (44.1)
Other	10 (2.1)	1 (1.2)	2 (1.7)	4 (2.5)	3 (2.5)

*LNs* Lymph nodes, *PD-L1* Programmed cell death ligand-1

^a^Multiple selection

^b^If the tumor location is the upper lobe of lung and/or level 2 LN station involved

^c^If the tumor location is left lung

## Discussion

The role of PORT as an adjuvant treatment in completely resected pIIIA-N2 NSCLC patients is controversial, and incorporating radiation oncologists’ opinions is of paramount importance. In our study, we conducted a national survey across Chinese radiation oncologists and found that most of the respondents replied that the decision of whether to administer PORT should be made on an individual basis for each patient after the publication of the two RCTs. Meanwhile, the proportion of respondents recommending PORT was significantly decreased after the publication of the two RCTs, suggesting that most would agree that PORT did not confer OS benefits to pIIIA-N2 NSCLC patients. Therefore, the negative results not only led to the updating of some guidelines [14] but also affected respondents’ treatment decisions. However, the proportion of Chinese radiation oncologists not recommending PORT increased only slightly, which means that a significant majority agreed that PORT should not be completely abandoned. This is consistent with the recent NCCN guideline [15] that PORT should be implemented tailoring to individual risk profiles rather than completely discarding it.

Some substantial information might contribute to this status. First, inspiring results related to LRR, DFS and OS from previous retrospective studies and meta-analyses prevent us from avoiding PORT. Second, although DFS in the PORT group was not significantly improved compared with that in the group without PORT, a slight advantage could be found in both trials (Lung ART: 30.5 months vs. 22.8 months, hazard ratio [HR] = 0.86, *p* = 0.180; PORT-C: 22.1 vs. 18.6 months, HR = 0.84, *p* = 0.200) [10]. Third, the most common PORT technique used in the Lung ART was three-dimensional conformal radiotherapy (3D-CRT), with a prevalence of 89%, rather than the more advanced IMRT technique, which may increase radiotherapy toxicity [10]. Additionally, there were no uniform dose constraints for normal tissues, and the total radiotherapy dose of 54 Gy was higher. For the PORT-C trial, the number of modified intent-to-treat patients was 364, while the number of per-protocol patients was only 310, which revealed that the compliance of the enrolled patients in the study was relatively low [11]. Thus, the generalizability of the results may be compromised due to the above limitations. Instead, the proportion of respondents who made the decision based on risk factors was largely increased (from 33.1 to 69.0%). Süveg et al. conducted an investigation of decision-making related to PORT before and after presentation of the results of the Lung ART among 22 European experts [16]. Their findings are consistent with ours—the majority of radiation oncologists (82.0%) recommended PORT for pIIIA-N2 patients with risk factors [16].

Identifying risk factors would allow radiation oncologists to personalize PORT for patients based on their risk levels, which could further reduce the toxicity and improve survival. Therefore, the current research aimed to identify several high-priority risk factors that influence the decision-making related to PORT. In our study, we identified the 5 most important risk factors: nodal extracapsular extension, the highest LN station involved, the number of dissected mediastinal LN stations, the number of positive mediastinal LN stations, and surgical approaches. These factors should be considered in future studies. Nevertheless, there is still a lack of prognostic scoring systems based on these high-priority risk factors to guide future studies to perform stratified analysis and administer PORT to suitable patients.

Previous studies investigating prognostic factors mainly focused on nodal involvement. Nodal extracapsular extension, defined as the occurrence of metastatic tumor cells extending through the LN capsule into the surrounding tissues, is known to be a negative predictor of local recurrence and survival in a variety of cancers [17–19]. In the Lung ART, patients without nodal extracapsular extension gained a significant 3-year mediastinal relapse-free benefit from PORT (HR = 0.46). Other retrospective studies also reported that nodal extracapsular extension is associated with DFS in pN2 patients [20–22]. The preplanned exploratory analysis in the PORT-C trial showed that patients at high risk for relapse, including the patients with > 20 dissected LNs or ≥ 4 positive LNs, had significantly shorter DFS. Another study found that PORT could significantly improve OS and decrease overall mortality in patients with ≥ 6 positive LNs [23]. Additionally, the ratio of positive LNs with a cutoff value of 50% was an independent risk factor for OS, which was consistent with the selection of most respondents in our study [24].

However, our research found that radiation oncologists were biased toward the LN stations, and the highest LN station involved as well as the number of dissected and positive mediastinal LN stations were considered more important than the LNs themselves according to how many times these risk factors were selected. This may be because the individual differences in the number of LNs found and removed are relatively large compared with LN stations. Wei W et al. reported that multiple LN station involvement was associated with short local recurrence-free survival (LRFS) and OS, suggesting the significance of the status of LN station in terms of prognosis [25]. It was noted that multiple LN station involvement occurred in only approximately one-third of patients in Lung ART trial, which means that the majority of patients in this study might be in the low-risk group and thus could not gain additional benefit from PORT. In addition to LN-related risk factors, the surgery method was also one of the 5 most considered risk factors. There is evidence that the type of surgery was an independent prognostic factor [26]. Additionally, recent studies suggested that detecting MRD was useful for identifying patients with a high risk of relapse by ctDNA analysis, thereby contributing to the personalization of adjuvant therapies [27–29]. Postsurgical ctDNA-positive lung cancer patients were significantly associated with poor recurrence-free survival and could benefit from adjuvant chemotherapy [28]. Moreover, the application of PORT might lead to ctDNA clearance, which indicates that PORT could serve as an effective therapeutic method to eliminate MRD and thus improve outcomes [29].

Heterogeneity among radiotherapy techniques might result in inconsistent results among studies of PORT. In 1998, a large meta-analysis showed that in completely resistant NSCLC with stage III and N2 disease, PORT did not provide additional survival benefits but did improve local control; however, old radiotherapy techniques such as two-dimensional (2D) radiotherapy were used[5]. A subsequent study reported that PORT might be associated with both improved local control and improved survival due to the wide application of modern radiotherapy techniques, which largely reduce treatment-related toxicity [30]. The radiotherapy techniques used in the Lung ART and PORT-C trial were also different, which could explain differences in the rates of toxicity. The most commonly used radiotherapy technique used in the Lung ART was 3D-CRT, while IMRT was the most common technique in the PORT-C trial. Thus, radiotherapy toxicity appears more frequent and severe in the Lung ART compared with PORT-C (grade 2 or higher radiation pneumonitis: 50.7% vs. 36.6%, grade 3 or lower radiation esophagitis: 19.0% vs. 6.0%) [10, 11]. In addition, the radiotherapy dose, CTV, and dose restrictions to the organs at risk also had an effect on the level of toxicity to some extent. Therefore, our survey investigated the radiotherapy-related factors that are often used in the clinical application of PORT among Chinese radiation oncologists. A total of 68.2% of respondents reported that IMRT could be applied to treat patients in their institutions, and the total dose of 50 Gy was selected in the majority of them. Lung stump + ipsilateral hilus + regions containing positive LNs were the most frequent CTV used by respondents. For dose restrictions to the organs at risk, lung V20 less than 25%, heart V30 less than 40%, and heart V40 less than 30% were mostly recommended. These parameters provide a reference for subsequent clinical trials to reduce the radiotherapy toxicity of PORT.

This study had some strengths. To our knowledge, this is the largest study of expert opinions on PORT and related risk factors. Nearly 500 Chinese radiation oncologists from 29 provinces participated in the survey with wide geographic coverage and a relatively large sample; thus, our data are nationally representative. Additionally, the study was also more comprehensive than previous studies in terms of the risk factors examined and treatment details of PORT. We divided risk factors into LN-related and non-LN-related risk factors and provided multiple options for participants to choose, and we aimed to determine appropriate cutoff values, which is likely to be useful as a reference for future trials.

This study also had limitations. First, respondents were self-selected; the number of radiotherapy oncologists who were exposed to the survey is unknown, and thus, self-selection bias is a concern. For instance, radiation oncologists who were more interested in PORT might have been more likely to respond. In addition, some radiation oncologists who used the internet infrequently might not have seen the survey. Thus, the descriptive statistics reported here may not fully reflect the opinions of all Chinese radiation oncologists. Second, approximately one-fourth of respondents did not know about the Lung ART and PORT-C trial, and this heterogeneity in exposure to information can influence respondents’ decision-making. Finally, our study is a cross-sectional survey in which data were all self-reported, thus leading to the potential for misclassification.

## Conclusion

Although routine use of PORT in completely resected IIIA-N2 NSCLC patients cannot be recommended, our findings suggest that most radiation oncologists make treatment decisions based on individual risk factors, especially LN status, rather than completely discarding the potential benefits of PORT in high-risk populations. Future prospective studies are necessary to define potential high-risk populations who can benefit from PORT treatment.

## Supplementary Information

**Additional file 1.** An overview of the questionnaire content.

## Data Availability

The datasets used and/or analyzed during the current study are available from the corresponding author on reasonable request.

## Abbreviations

NSCLC: Non-small cell lung cancer
LRR: Local-regional recurrence
PORT: Postoperative radiotherapy
DFS: Disease-free survival
PP: Per-protocol
OS: Overall survival
IQR: Interquartile range
GDP: Gross domestic product
IMRT: Intensity-modulated radiotherapy
LN: Lymph node
ctDNA: Circulating tumor DNA
MRD: Minimal residual disease
CTV: Clinical targeted volume
RCTs: Randomized clinical trials
3D-CRT: Three-dimensional conformal radiotherapy
HR: Hazard ratio
LRFS: Local recurrence-free survival
2D: Two-dimensional
