# Supplementary material for: Real world practice of postoperative radiotherapy for patients with completely resected pIIIA-N2 non-small cell lung cancer: a national survey of radiation oncologists in China
Source: Radiat Oncol. 2023 Jan 25;18:17. doi: 10.1186/s13014-023-02208-5 (PMC9875489; doi:10.1186/s13014-023-02208-5)
Supplement: Supplementary file 1 — Additional file 1. An overview of the questionnaire content. [file 13014_2023_2208_MOESM1_ESM.docx]

A Survey of Postoperative Radiotherapy for Patients with Completely Resected pIIIA-N2 Non-small Cell Lung Cancer

Question 1. What’s your name?

_________________________________

Question 2. How old are you?

_________________________________

Question 3. What‘s your gender

_________________________________

Question 4. Which province, autonomous region or municipalitie do you live in?

_________________________________

Question 5. What’s the level of the hospital where you work? [single choice]

| ○Tertiary class A hospital |
| --- |
| ○Tertiary class B hospital |
| ○Secondary hospital |
| ○Other |

Question 6. What’s the type of the hospital where you work? [single choice]

| ○Comprehensive hospital |
| --- |
| ○Cancer specialty hospital |

Question 7. What’s your professional titles? [single choice]

| ○Senior title |
| --- |
| ○Intermediate title |
| ○Junior title |

Question 8. What’s your educational level? [single choice]

| ○Doctorate |
| --- |
| ○Master |
| ○Bachelor |

Question 9. What’s your clinical experience (defined as years practicing thoracic radiation)? [single choice]

| ○1-5 years |
| --- |
| ○6-10 years |
| ○11-20 years |
| ○≥ 21 years |

Question 10. Do you know the LungART study published on the journal of Lancet Oncology? [single choice]

| ○Yes | ○No |  |  |  |  |  |  |
| --- | --- | --- | --- | --- | --- | --- | --- |

Question 11. Do you know the PORT-C study published on the journal of JAMA Oncology? [single choice]

| ○Yes | ○No |  |  |  |  |  |  |
| --- | --- | --- | --- | --- | --- | --- | --- |

Question 12. Before LungART and PORT-C published, which of followings applies to your practice for completely resected IIIA-N2 NSCLC? [single choice]

○Recommend PORT ○ Not recommend PORT ○According to individual risk factors

Question 13. After LungART and PORT-C published, which of followings applies to your practice for completely resected IIIA-N2 NSCLC? [single choice]

○Recommend PORT ○ Not recommend PORT ○According to individual risk factors

Question 14. Which of followings about surgical approaches that patients receive you are more likely to recommend PORT? [multiple choice]

| □Not be a consideration |
| --- |
| □Pneumonectomy |
| □Lobectomy |
| □Sleeve lobectomy |

Question 15. Which of followings about margin distance you are more likely to recommend PORT? [single choice]

| ○Not be a consideration |
| --- |
| ○0.5cm |
| ○1cm |
| ○1.5cm |
| ○2cm |
| ○Other _________________ * (Please fill in the blank space if options are not provided) |

Question 16. Which of followings about pre-operative lymph node conditions you are more likely to recommend PORT? [single choice] [multiple choice]

| □Not be a consideration |
| --- |
| □Multiple mediastinal lymph node stations involved |
| □The largest short diameter of positive mediastinal lymph node ≥2cm |
| □Multiple positive mediastinal lymph nodes fused |
| □Lymph nodes invading the surrounding tissue on imaging |
| □Other _________________ (Please fill in the blank space if options are not provided) |

Question 17. Do you evaluate the nodal extracapsular extension? [single choice]

| ○Yes |
| --- |
| ○No |
| ○Sometimes |

Question 18. If patients have nodal extracapsular extension, are you more likely to recommend PORT for them? [single choice]

| ○Not be a consideration |
| --- |
| ○Yes |
| ○No |
| ○Sometimes |

Question 19. If patients have the highest lymph node station involved, are you more likely to recommend PORT for them? [single choice]

| ○Not be a consideration |
| --- |
| ○Yes |
| ○No |
| ○Sometimes |

Question 20. Which of followings about the number of resected LNs you are more likely to recommend PORT? [single choice]

○Not be a consideration ○≤10 ○≤11 ○≤12 ○≤13 ○≤14 ○≤15 ○≤16

Question 21. Which of followings about the number of resected mediastinal LNs stations you are more likely to recommend PORT? [single choice]

○Not be a consideration ○≤1 ○≤2 ○≤3

Question 22. Which of followings about the number of positive LNs you are more likely to recommend PORT? [single choice]

○Not be a consideration ○≥1 ○≥2 ○≥3 ○≥4 ○≥5 ○≥6

Question 23. Which of followings about the number of positive mediastinal LNs stations you are more likely to recommend PORT? [single choice]

○Not be a consideration ○≥1 ○≥2 ○≥3

Question 24. Which of followings about the number of positive LNs ratio you are more likely to recommend PORT? [single choice]

○Not be a consideration ○≥10% ○≥20% ○≥30% ○≥40% ○≥50%

Question 25. In addition to the above-mentioned factors, which LNs-related factors you consider in the clinical practice? [fill-in-the-blank]

_________________________________

Question 26. Which of followings about the pT stage (based on AJCC 8th edition) you are more likely to recommend PORT? [single choice]

○Not be a consideration ○≥1b ○≥1c ○≥2a ○≥2b ○≥3 ○≥4

Question 27. If patients have received preoperative neoadjuvant treatment with cN2 stage, which of followings about pN stage you are more likely to recommend PORT? [multiple choice] *

| □ypN0 |
| --- |
| □ypN1 |
| □ypN2 |
| □Recommend PORT regardless of the pN stage |
| □Not recommend PORT regardless of the pN stage |

Question 28. If patients have positive driver gene mutations, which of followings about adjuvant therapeutic modalities after surgery you are more likely to recommend PORT? [multiple choice] *

| □No adjuvant therapy |
| --- |
| □Chemotherapy |
| □Targeted therapy |
| □Chemotherapy + Targeted therapy |
| □Recommend PORT regardless of the therapeutic modalities |
| □Not recommend PORT regardless of the therapeutic modalities |

Question 29. If patients have positive PD-L1 expression, which of followings about adjuvant therapeutic modalities after surgery you are more likely to recommend PORT? [multiple choice] *

| □No adjuvant therapy |
| --- |
| □Chemotherapy |
| □Immunotherapy |
| □Chemotherapy + Immunotherapy |
| □Recommend PORT regardless of the therapeutic modalities |
| □Not recommend PORT regardless of the therapeutic modalities |

Question 30. Which of followings about ECOG PS you may consider the possibility to administer PORT? [single choice] *

| ○Not be a consideration |
| --- |
| ○≤0 |
| ○≤1 |
| ○≤2 |
| ○≤3 |
| ○≤4 |

Question 31. Which of followings about age you may consider the possibility to administer PORT? [single choice] *

| ○Not be a consideration |
| --- |
| ○≤60 |
| ○≤70 |
| ○≤75 |
| ○≤80 |

Question 32. In addition to the above-mentioned factors, which risk factors you consider in the clinical practice?？ [multiple choice]

| □The lung function |
| --- |
| □The heart function |
| □History of current or previous smoking |
| □Whether underwent a baseline PET–CT |
| □Whether ctDNA existed upon tumor resection. |
| □Whether intravascular cancer thrombus existed |
| □The distance between the patient’s home and radiotherapy center |
| □Other _________________ (Please fill in the blank space if options are not provided) |

Question 33. What’s your recommended dose of PORT? [single choice]

| ○50Gy, conventional segmentation |
| --- |
| ○54Gy, conventional segmentation |
| ○Other _________________ (Please fill in the blank space if options are not provided) |

Question 34. Which of followings about standard technique for PORT you usually use in your hospital? [single choice] *

| ○2D RT |
| --- |
| ○3D CRT |
| ○IMRT |
| ○VMAT |
| ○TOMO |
| ○MR-Liac |
| ○Proton/heavy ion |

Question 35. Which targeted region of PORT is closest to your usual practice? [multiple choice] *

| □Lung stump + ipsilateral hilus + regions containing positive LNs |
| --- |
| □Lung stump + ipsilateral mediastinum ± ipsilateral supraclavicular LNs (if tumor in the upper lobe of lung and/or level 2 LNs station involved) |
| □Lung stump + ipsilateral mediastinum ± contralateral upper mediastinum (if primary tumor in the left lung) |

Question 36. If PORT is planned, under which circumstance you will implement prophylactic radiotherapy for stump? [multiple choice] *

| □Surgical margins were near to tumor margins |
| --- |
| □Central type |
| □Any condition |

Question 37. If PORT is planned, under which circumstance you will implement prophylactic radiotherapy for supraclavicular region [multiple choice] *

| □Positive mediastinal LNs at level 2 |
| --- |
| □Other _________________ (Please fill in the blank space if options are not provided) |

Question 38. If PORT is planned, under which circumstance you will implement prophylactic radiotherapy for contralateral mediastinum [multiple choice] *

| □Primary tumor in the left lower lobe |
| --- |
| □Positive LNs ratio of 100% at level 7 |
| □Other _________________ (Please fill in the blank space if options are not provided) |

Question 39. If patients have received lobectomy, what’s the dose constraint of the lungs V20 in your clinical practice? [single choice] *

| ○<20% |
| --- |
| ○<25% |
| ○<30% |
| ○Other _________________ (Please fill in the blank space if options are not provided) |

Question 40. What’s the dose constraint of the heart in your clinical practice? [multiple choice] *

| □No limitation |
| --- |
| □Yes, Heart V30 < _________________ |
| □Yes, Heart V40 < _________________ |

Question 41. If patients tolerate PORT, please select the five most important risk factors influence your decision-making of PORT from the following options [multiple choice] *

| □Evaluated by PET-CT |
| --- |
| □Surgery methods |
| □Nodal extracapsular extension |
| □Highest LNs station involved |
| □The number of dissected LNs |
| □The number of dissected mediastinal LNs stations |
| □The number of positive LNs |
| □The number of positive mediastinal LNs stations |
| □Positive LNs ratio |
| □pT stage |
| □ctDNA status |
| □Portal vascular invasion |
| □Adjuvant targeted therapy |
| □Adjuvant immunotherapy |
| □Radiotherapy techniques |
| □Transportation convenience |
| □Other _________________ (Please fill in the blank space if options are not provided) |
